# Implicit bias assessment by career stage in medical education training: a narrative review

**DOI:** 10.1186/s12909-024-06319-9

**Published:** 2025-01-28

**Authors:** Alisha Crump, May Saad Al-Jorani, Sunya Ahmed, Ekas Abrol, Shikha Jain

**Affiliations:** 1 School of Pharmacy, University of Maryland, Postdoctoral Fellow, Baltimore, MD US; 2 College of Medicine, Medical Student, Mustansiriyah University, Baghdad, Iraq; 3 St. George’s University, School of Medicine West Indies, Medical Student, West Indies, Grenada; 4 The University of Illinois Cancer Center, Research Specialist, Chicago, IL US; 5 University of Illinois Chicago, College of Medicine, Associate Professor of Medicine, Chicago, IL US

**Keywords:** Implicit bias, Medical education, Pre-medical, Medical, Graduate

## Abstract

Implicit biases involve associations outside conscious awareness that lead to a negative evaluation of a person based on individual characteristics. Early evaluation of implicit bias in medical training can prevent long-term adverse health outcomes related to racial bias. However, to our knowledge, no present studies examine the sequential assessment of implicit bias through the different stages of medical training. The objective of this narrative review is to examine the breadth of existing publications that assess implicit bias at the current levels of medical training, pre-medical, graduate, and postgraduate. Protocol for this study was drafted using the Scale for the Assessment of Narrative Reviews (SANRA). Keyword literature search on peer-reviewed databases Google Scholar, PubMed, Ebsco, ScienceDirect, and MedEd Portal from January 1, 2017, to March 1, 2022, was used to identify applicable research articles. The online database search identified 1,512 articles. Full screening resulted in 75 papers meeting the inclusion criteria. Over 50% of extracted papers (74%) were published between 2019 and 2021 and investigated implicit bias at the post-graduate level (43%), followed by the graduate level (34%), and pre-medical level (9.4%). Fourteen percent were classified as mixed. Studies at the medical and medical graduate level identified an implicit preference towards white, male, non-LGBTQIA+, thin, patients. Study findings highlight notable gaps within the sequential assessment of implicit bias, specifically at the pre-medical training level. Longitudinal epidemiological research is needed to examine the long-term effect of implicit biases on existing healthcare disparities.

## Background

Implicit bias involves unconsciousassociations resulting in negative evaluations of a person based on individual characteristics such as race, gender, sexual orientation, and religion [1]. Several mechanisms contribute to the formation of harbored implicit biases. Current research has identified two pathways involved in this action. Type 1 decision-making is fast, unconscious, intuitive, and involves "mental shortcuts or heuristics", while type 2 is a slower, 'analytical' process that requires higher cognitive ability [2, 3]. The resulting individual behavioral phenomena, a combination of type 1 and type 2 decision-making styles, can be affected by various experienced factors, such as implicit bias [4]. Through exposure to stereotypes and misinformation, implicit bias can take root, impacting personal and professional interactions. Unlike explicit biases, which involve attitudes and assumptions that we acknowledge, implicit bias can surreptitiously influence judgment through the lack of individual intent: a person can displaya dissociation between explicit attitudes and implicit associations [5]. Without proper mitigation, over time, these unacknowledged biases can result in microaggressions, prejudice, and the further marginalization of communities.

The cause of implicit bias is among themany contributing factors that lead to health inequity and has resulted in a remarkable effect on clinician diagnoses, bedside treatment, and patient consultations [6]. Results of a 2015 systematic review showed that most healthcare clinicians have an implicit bias against people of color, including Black, Hispanic/Latine and dark-skinned individuals [7]. The result of discriminatory behavior within healthcare has led to poor patient outcomes such as reduced medication adherence, and a discontinuity of care, specifically among marginalized racial groups [8, 9]. 

Racial bias within healthcare is merely one example of implicit assumptions held within the field. Weight bias has also demonstrated similar findings, with many healthcare clinicians harboring strong negative attitudes and stereotypes toward obese or overweight patients [10, 11]. Gender blindness and preconceived stereotypes about men and women are contributing factors to gender-specific biases [12]. These distortions are dramatized for specific health conditions, like myocardial infarction in which women often present with 'atypical' chest pain such as nausea, vomiting, and palpitations [4, 13]. Furthermore, while race, weight, and gender are the most researched types of implicit bias, several others exist, such as sexual orientation and religious preferences.

The multifactorial nature of implicit bias presents ongoing challenges and has resulted in limited detection methods in public health and clinical settings [14]. The Harvard Implicit Assessment test (IAT) is the most commonly used method to identify implicit bias [15, 16]. Although this test is widely used in current literature, opponents of the IAT have highlighted several limitations such as a lack of measurement accuracy and poor differentiation between association and automatically activated responses [17]. Out of this uncertainty, many other methods have emerged such as focus groups, and the establishment of academic curricula. However, such biases exist within larger economic and social structure perpetuated by systemic prejudice. As a result of this complexity hidden within the intrinsic nature of implicit biases, research on this topic has been limited.

### Implicit bias in medical education training

The prevalence of implicit bias among clinicians can be partly attributed to unaddressed discriminatory experiences throughout life and the perpetuation of these experiences within medical education settings [5, 18]. A 2015 longitudinal study reported that 48.7% of U.S. medical students were exposed to negative comments about Black patients by attending or resident physicians [5]. As a result, these students experienced significantly greater implicit racial bias in year 4 compared to year 1 [5]. Based on these results, and many supporting others, the impact of implicit bias and adverse health outcomes at the level of physician can be mitigated by experiences during medical education [19–22]. However, there is sparse knowledge about the implementation of implicit bias training programs within the medical education path. Furthermore, no present study to our current knowledge has examined the sequential assessment of implicit bias through the different stages of medical training, beginning at the pre-medical level. To address this gap, the objective of the current study is to perform a narrative review to evaluate the breadth of existing publications that assess implicit bias at the different levels of medical training, pre-medical, graduate, and post-graduate.

## Methods

A narrative review approach was used to assess implicit bias training throughout medical education. In theory, narrative reviews can be useful in identifying research gaps and critical areas where stronger policy and practice is necessary. The narrative review was guided by the research criterion: 1) The stage of medical education at which implicit bias was assessed [pre-medical, graduate, medical graduate], and 2) the specific methods used to measure implicit bias at each educational level.

### Literature search

To maintain the efficacy and systematic nature of our study, two reviewers (A.C., and M.S.) generated a search string of terms associated with implicit bias and medical education. Search terms were identified as descriptive of implicit bias and related to required medical education [pre-medical, medical, and medical graduate].

The initial search was conducted on February 26, 2022, on six selected research databases: Google Scholar, PubMed, Ebsco, MedPortal, Scopus, and ScienceDirect. Databases were selected broadly to cover the breadth of implicit bias interpretations and information on medical educational training. No study design restrictions were applied. The total number of collected studies from each search engine was validated to ensure the full scope of available articles was collected. All articles collected during the literature search were imported into Zotero, a standardized reference management software. The search was completed on March 14, 2022, and updated on March 27, 2022, to include any additional newly published articles since the completion date.

### Inclusion criteria

Two reviewers independently screened and applied inclusion criteria to the collected articles gathered from the literature search (A.C. and M.S). To examine the published articles before and during the impact of COVID-19, the years 2017–2022 were included in the study. Collected articles from the initial search were halved and inclusion criteria were applied between two reviewers (A.C. and M.S.). Titles and abstracts were initially screened for the scope and relevance of the research aim. All discrepancies were resolved from team consensus.

Studies were identified as those at the pre-medical level if they included a population of students at the undergraduate level with an intended focus on medical school attendance after graduation. Those studies at the medical and graduate level included students currently enrolled in medical school and at the level of resident, respectively. Since not all medical students enroll in clinical fellowships, publications including this subgroup were not included in the article search as these articles were not within the scope of the proposed study’s research question. Assessment of implicit bias at individual levels of medical education was identified as having examined the presence of implicit bias by Implicit Association Tests, social focus groups, and other assessment methods described elsewhere [23].

Studies were included if they assessed implicit bias at any level of medical education within the study’s research aim. Additional inclusion criteria included: 1) Full text was provided, 2) Published between 2017–2022, 3) Conducted in the United States, 4) English, 5) involved qualitative, quantitative, or mixed methods study design, and 6) within the scope of implicit bias and medical educational training.

### Data extraction, quality assessment, and analysis

Data was extracted using a standardized protocol in Microsoft Excel. Extracted data included authors, year, medical training level, implicit bias assessment method, category of implicit bias explored (i.e. race, gender, socioeconomic status, etc.), study results, and conclusion. Articles extracted among one reviewer (A.C. or M.S.) were reviewed by the other reviewer for verified accuracy and completeness. Methodological quality was assessed using the Assessment of Scale for the Assessment of Narrative Reviews [24]. The Assessment of Scale for the Assessment of Narrative Reviews (SANRA) is a methodological tool used to evaluate the quality of narrative review articles, by assessing things like research aims, study methodology and presentation of evidence [24]. Discrepancies in manuscript assessment were resolved through discussion and consensus between the two reviewers (A.C. and M.S.).

## Results

**Fig. 1:**
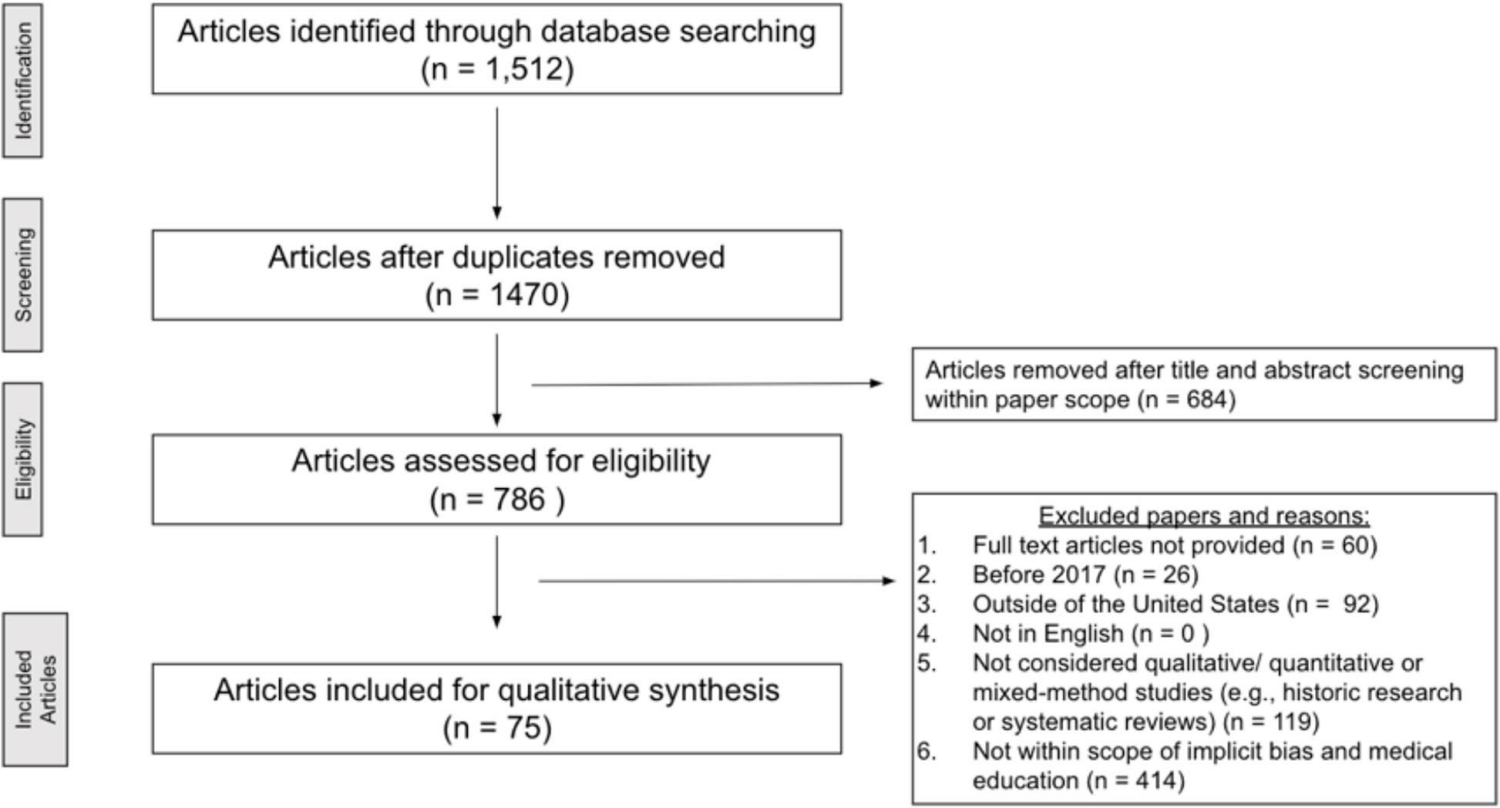
Article screening and eligibility flowchart

Figure 1 demonstrates the flowchart for article eligibility. A total of 1,512 articles were identified through database searching, with 42 duplicates removed; 1,470 articles were then screened based on the title, abstract, and relevance to the study objective. Five literature review articles were included due to their breadth of implicit bias training methods and discussion. The screening step resulted in 786 articles being assessed for eligibility. Of those 786 articles, 711 were excluded, leaving 75 articles included in the qualitative synthesis.

Table 1 lists the included studies that met study eligibility criteria. Over 50% of articles were published between 2019–2021. In 2022, only two published articles targeted implicit bias within medical education, a decrease potentially explained by our data extraction timeline and not a lack of topic significance during this period. Implicit bias was least assessed at the pre-medical education level, with approximately 50% of the total articles focusing on implicit bias as a general concept (non-specific to any form of bias). Four articles explored intersectional forms of implicit bias, such as the combined effects of socioeconomic and occupational status.

**Table 1:** List of all manuscripts that met eligibility criterion

Authors	Year	Training Level	Implicit Bias Assessment Method	Type of Implicit Bias Explored
Minturn et al. [25]	2021	Pre-Medical	10-h LGBTQ health curriculum	LGBTQIA +
Petty et al. [26]	2017	Pre-Medical	Structural Foundations of Health Survey	Race
Metzl et al. [27]	2018	Pre-Medical	Structural Foundations of Health Survey	Race
Goyal et al. [28]	2020	Pre-Medical	Structured in-depth interviews	Cultural/Social
Munk et al. [29]	2020	Pre-Medical	Survey Design	Gender
Copeland et al. [30]	2020	Pre-Medical	Pre/Post Questionnaire	Socioeconomic Status
Martinez et al. [31]	2022	Pre-Medical	Skills and Knowledge based interventions	Attitude / Reflection
Gonzalez et al. [20]	2021	Medical Student	1-h, multimedia, interactive lecture to all first-year medical students	General
Baker et al. [32]	2017	Medical Student	Implicit Relational Assessment Procedure	Weight
Pettit et al. [33]	2017	Medical Student	high-fidelity simulation of acute coronary syndrome	Socioeconomic Status
Phelan et al. [34]	2017	Medical Student	Web-based survey	LGBTQIA +
Sawning et al. [35]	2017	Medical Student	pretest-post-test design	LGBTQIA +
Geller et al. [36]	2018	Medical Student	Ethics Education (Survey + Education course + IAT)	Weight
Lawrence et al. [37]	2018	Medical Student	NA (literature review of hidden curriculum)	General
Leslie et al. [38]	2018	Medical Student	Health Equity Curriculum Intervention	Race, Weight, LGBTQIA +
Marion et al. [39]	2018	Medical Student	Seminars + Patient Assessments	General
Gonzalez et al. [40]	2019	Medical Student	Focus Groups	General
Horst et al. [41]	2019	Medical Students	Selecting and Performing Service prompts aimed at self-examination, bias mitigation, and compassionate behavior	General
Motzkus et al. [42]	2019	Medical Student	IAT + Determinants of Health Course	General
Phelan et al. [43]	2019	Medical Student	Survey	Race
Acholonu et al. [44]	2020	Medical Student	Academic Workshop	General
Benoit et al. [45]	2020	Medical Student	A student-led initiative (Curriculum + Guidelines + Existing learning environment)	General
Gonzalez et al. [46]	2020	Medical Student	Course intervention + Program evaluation	General
Morris et al. [47]	2020	Medical Student	Survey	Socioeconomic Status, LGBTQIA + , Occupation
Ona et al. [48]	2020	Medical Student	Antiracism Curriculum	Race
Rivlin et al. [49]	2020	Medical Student	Open-ended written questionnaire	Medical Decisions (abortion)
Ruben et al. [50]	2020	Medical Student	Three-Part Implicit Bias Training Program	Skin Tone
Fitterman et al. [51]	2021	Medical Student	Curriculum-based intervention	Weight
Gonzalez et al. [52]	2021	Medical Student	One 90-min session on implicit bias	General
Gonzalez et al. [53]	2021	Medical Student	Skills-Based Curriculum	General
Nestorowicz et al. [54]	2021	Medical Student	Beliefs About Obese Persons Scale, Attitudes Toward Obese Persons Scale, Fat Phobia Scale, and the Harvard Implicit Association Test (IAT) and researcher-generated questions, measured levels of bias before and after study activities	Weight
Phelan et al. [55]	2021	Medical Student	Survey + Patient Care Scenario	Weight
Van Winkle et al. [56]	2021	Medical Student	Medical Humanities Course	General
Matsumato et al. [57]	2020	Medical Student	Survey	Gender
Chen et al. [58]	2021	Medical Students	Evaluations	Gender
Gopal et al. [59]	2021	Mixed	Mixed (literature review)	General
Fassioto et al. [60]	2018	Mixed (residents and fellows)	Evaluations	Gender
Halvorson et al. [61]	2019	Mixed (pediatric hospitalists, residents, and acute care nurses)	- Semi-structured Interviews, - IAT (implicit) and - Crandall's Anti-Fat Attitudes Questionnaire (explicit)	Weight
Morris et al. [62]	2019	Mixed (medical, dental and nursing students)	Mixed (literature review) - including programs, experimental interventions, etc	LGBTQIA +
Brottman et al. [63]	2020	Mixed (medical students and professional)	Educational strategies, Academic curriculum	General
Mastrocola et al. [64]	2020	Mixed (residents and fellows)	Obesity education programs	Weight
Teherani et al. [65]	2020	Mixed (Medical Students and Residents)	Semi structured interviews	General
Tobon et al. [66]	2021	Mixed (Medical students and psychiatric physicians)	IAT	Race
Ogunyemi et al. [67]	2021	Mixed (Medical students, residents and faculty)	Structured, Interactive Workshop	Mixed (Gender, race, appearance (tattoos))
Xiong et al. [68]	2022	Mixed (faculty, fellows, residents, and medical students)	Survey (Likert-scale)	Gender
Bartlett et al. [69]	2019	Medical Graduate (Medical Trainees)	Workshop	General
Kallianos et al. [70]	2019	Mixed (Faculty and Trainees)	IAT and Survey	General
Sherman et al. [71]	2019	Mixed (Faculty and Trainees)	Trainings and Focus Groups	General
Herr et al. [72]	2020	Mixed (Medical students and post medical trainees)	N/A (Literature Review)	General
Perdomo et al. [73]	2019	Medical Graduate (Residents)	Health Equity Rounds Curriculum	General
Barnes et al. [74]	2019	Medical Graduate (Surgical Trainees)	Focus Groups	Gender
Johnson et al. [75]	2017	Medical Graduate (Residents)	Implicit Association Tests (IATs)	Race
Kulayat et al. [76]	2017	Medical Graduate (Trainees)	Small group discussions/ Focus groups	General
Chapman et al. [77]	2018	Medical Graduate (Medical Trainees)	Intervention focus Groups	Race/ Ethnicity
Bucknor et al. [78]	2019	Medical Graduate (Medical Trainees)	Verbal Responses	Weight
Dyrbye et al. [79]	2019	Medical Graduate (Medical Trainees)	Questionnaire	Race
Gerull et al. [80]	2019	Medical Graduate (Surgical Trainees)	Written Evaluations	Gender
Hansen et al. [81]	2019	Medical Graduate (Resident Physicians)	Survey	Gender
Khatri et al. [82]	2019	Medical Graduate (Medical Trainees)	IAT and Facilitated Discussion	General
Klein et al. [83]	2019	Medical Graduate (Medical Trainees)	N/A (Literature Review)	Gender
Lukela et al. [84]	2019	Medical Graduate (Medical Trainees)	Cross-Sectional Survey	Gender
McKinley et al. [85]	2019	Medical Graduate (Medical Trainees)	Survey	Gender
Wittlin et al. [86]	2019	Medical Graduate (Medical Trainees)	Survey	LGBTQIA +
Zeidan et al. [87]	2019	Medical Graduate (Medical Trainees)	IAT and Curriculum Intervention	General
Kassam et al. [88]	2020	Medical Graduate (Medical Trainees)	Resident Applications	General
Klein et al. [89]	2020	Medical Graduate (Medical Trainees)	Intervention and Cross-Sectional Surveys	Gender
Kuo et al. [90]	2020	Medical Graduate (Surgical Trainees)	Templated Spreadsheet	Gender
Sabin et al. [91]	2020	Medical Graduate (Medical Trainees)	IAT + Intervention	General
Thomas et al. [92]	2020	Medical Graduate (Medical Trainees)	Curriculum Changes	Race and General
Barber et al. [93]	2020	Medical Graduate (Medical Trainees)	Cross-Sectional Survey	General
Chatterjee et al. [94]	2020	Medical Graduate (Medical Trainees)	Gender Bias Curriculum	Gender
Dill-Macky et al. [95]	2021	Medical Graduate (Surgical Trainees)	Evaluations	General
Kramer et al. [96]	2021	Medical Graduate (Medical Trainees)	Implicit Assessment Test (IAT)	Gender
Ouyang et al. [97]	2021	Medical Graduate (Surgical Trainees)	Interview Survey	Gender
Roth et al. [98]	2021	Medical Graduate (Medical Trainees)	Questioning Health Curriculum	LGBTQIA +

**Fig. 2:**
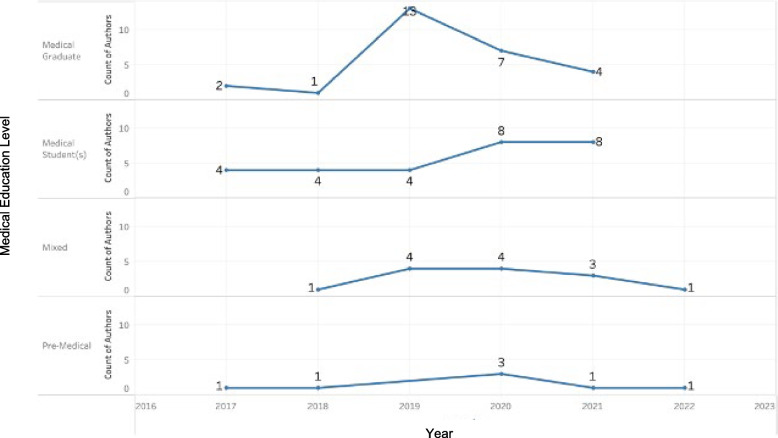
Graph by year and number of published articles at the different levels of medical education

Figure 2 demonstrates the annual number of published articles at each level of medical education from 2017 – 2022. From 2017—2018, the number of published articles remained nearly constant from the previous year among all levels of medical education. However, between 2018 and 2019, the number of published articles assessing implicit bias increased at the medical graduate, mixed, and pre-medical levels. Findings remained constant in the previous year among current medical students. In the years 2019–2020, published articles on the pre-medical and medical student educational level increased while mixed and medical graduate, remained constant and decreased, respectively. From 2020–2021, published articles on the study’s objective decreased among the pre-medical, mixed, and medical graduate educational levels. This pattern continued for the mixed category, while pre-medical remained constant. Published papers were not available in the 2022 data extraction for the medical graduate and medical student education levels.

### Definition of implicit bias

The definition of implicit bias was relatively consistent across all included articles. Most articles defined implicit bias as an unrecognized negative attitude towards a specific patient population (such as weight, race, gender, or sexual orientation) unless discussed in a general context. As an introduction to the concept, implicit bias was contrasted with explicit behavior to highlight the impact within healthcare and the need for deeper research on the topic.

### Implicit bias assessment method

All studies identified implicit bias as a concern along the continuum of medical education in the United States. Using a sample of 2nd-year medical students, a study conducted by Leslie et. al using the IAT identified LGBTQIA + , race, and weight-associated implicit biases pre-curriculum intervention [38]. Observed biases persisted even after curriculum intervention; however, minor improvements were seen. Assessment methods varied by study; however, most studies identified the IAT as a foundational tool for implicit bias assessment used frequently in studies exploring the topic. Some studies used a mixed intervention design, including two or more implicit bias assessment measures (most frequently employing a qualitative and quantitative assessment), suggesting possible insufficiency of using IAT alone as the primary assessment tool.

**Fig. 3:**
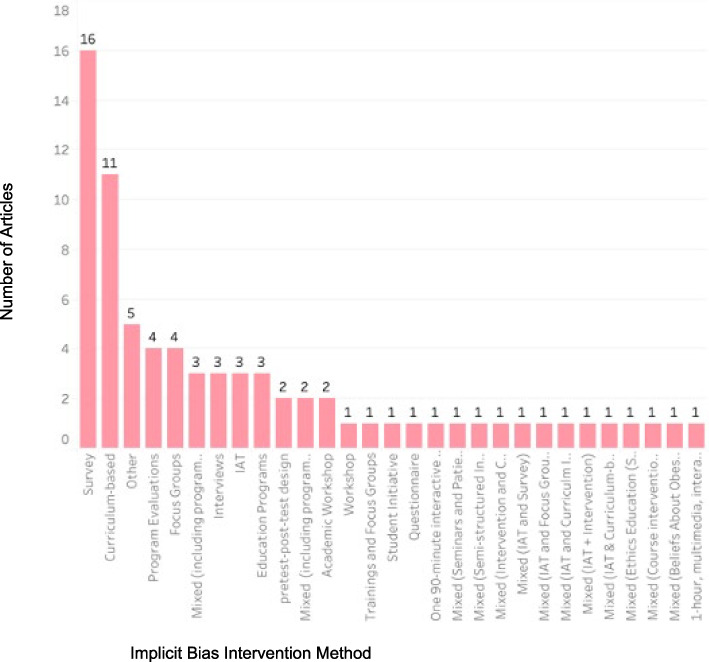
Graph by number of articles and assessment method

Figure 3 highlights the most common assessment methods mentioned among the extracted articles of the study. Surveys and curriculum-based approaches were the most common assessment tools, representing 21.3% and 14.7% of the published literature, respectively. IAT was used in less than 10% of articles. Thirteen studies used mixed methods such as IAT and focus groups/curriculum-based tools.

## Common forms of implicit bias

Research from 2017 to 2022 consistently revealed prevalent implicit biases in medical education, particularly regarding race, sexual orientation, gender, and weight. Overall, results at the medical student and medical graduate level suggest an implicit preference towards a male, non-LGBTQIA + , white, thin patients [10, 22, 25, 34, 99]. Hemphill et al. [100] recognized the persistence of unconscious or unintentional gender biases in medical education, and suggested that evidence-based guidelines tailored for graduate medical education are necessary to mitigate such occurrences [100]. This acknowledgment of pro-bias behavior aligns with prior research among medical students, which found that approximately 50% of students reported having negative attitudes towards lesbian and gay people, while about 80% harbored implicit biases [62, 99]. Expanding on these findings, a study conducted by Sabin et. al examined implicit and explicit racial attitudes among physicians and found that most physicians across all racial and ethnic groups demonstrated implicit bias favoring White Americans relative to Black Americans, among various specialties [22]. Furthermore, the study also revealed that female physicians and Black physicians, on average, exhibited lower levels of implicit racial bias compared to their male and non-Black counterparts, respectively [22]. In a separate longitudinal study of 4,732 participants, Phelan et al. [10] further demonstrated persistent anti-fat bias among first year medical students [10]. Collectively, these findings underscore the pervasive nature of implicit bias in medical education and the challenges in addressing it effectively. Notably, cultural bias was the least assessed form of implicit bias in our study, suggesting a potential area for future research.

## Potential solutions to implicit bias in medical training

Several studies discussed potential solutions to implicit bias throughout medical educational training. Solutions for implicit bias in medical training ranged broadly depending on the discussed bias, with many focusing on role-playing, reading, and reflection to demonstrate patient perspectives and highlight necessary skills to address bias in a clinical setting [101]. Reflective activities combined with discussions fostered a deeper understanding of information and allowed for the assessment of students’ understanding and awareness [25]. Role-playing exercises such as scripted interview exercises and training in sexual history taking have been instrumental in enhancing students’ comfort and confidence in working with LGBTQ patients [34, 102].

Collaborative approaches including educational training courses were also suggested as effective methods to reduce implicit bias. For biases associated with sexual orientation, specifically, the implementation of discussion-based courses was a helpful mechanism to increase knowledge about specific patient populations, such as the LGBT community [38]. Courses that incorporated discussions about the IAT and workshops on the social psychology of implicit bias have also been shown to reduce an implicit preference towards straight individuals [98]. Additionally, interactive presentations focusing on unconscious bias, discussions on intersectionality (i.e., the interconnectedness of social categorizations like race and gender), and visual demonstrations significantly raised perception and understanding of these biases and encouraged clinicians to ask sensitive history-related questions [63, 103]. In the classroom setting, assigned readings on cultural issues in healthcare further contributed to reducing students’ implicit bias by increasing awareness of interpersonal discriminator behaviors [25, 104].

## Discussion

Based on our findings, implicit bias was least studied among pre-medical students. Furthermore, articles that assessed implicit bias at the medical student and graduate levels suggest a preference for male, non-LGBTQIA + , white, thin patients. Inequality in patient preference further indicates that current implicit bias mitigation tools for current medical professionals are inadequate and may contribute to long-term health disparities. For example, unresolved implicit bias can in turn lead to confirmation or anchoring biases that cause a physician to prioritize their own inherent beliefs rather than alternative evidence and diagnoses. As a result, the misapplication of inherently well-intentioned practical theories, such as the social determinants, is used as justification for inequities in patient care, and increased prevalence of additional biases [104, 105].

The current study is novel in its inclusion of implicit bias throughout the process of medical education, a process often spanning well over a decade. By examining the topic across multiple educational levels, we offer a holistic perspective on the role of implicit bias along the student-to-clinician continuum. Limited research at the pre-medical level points to an opportunity for early intervention with implicit bias training that may impact medical professionals throughout their education and career. Simultaneously, documented persistence following standard training raises doubts about whether curriculum content adequately addresses the multifaceted biases spanning gender, racial, ageist, weight-based, and socioeconomic realms.

The current study has certain limitations. First, the exclusion of literature outside of the study period (2017–2022) results in a reduced perspective of implicit bias assessment and medical education. Second, as this study is primarily an assessment of the current literature, causality cannot be determined to examine how reduced assessment of implicit bias in medical education influences patient health outcomes. Third, the current study is restricted to published literature and does not include articles from other sources (i.e., grey literature); therefore, we cannot exclude the possibility of publication bias. Fourth, how prior studies have measured implicit bias may likely influence previous study findings and subsequent interpretations. Despite the limitations, the current study offers initial and impactful evidence on the assessment of implicit bias in the stages of medical education.

## Conclusion

The results of this review suggest that implicit bias is not assessed early in the medical education curriculum. Furthermore, these results highlight the need for a comprehensive and longitudinal approach to mitigating implicit bias in healthcare education. Future directions should be expanded to include examining implicit bias longitudinally across the span of medical education into later career medical training stages (i.e., attending physician status). The longitudinal examination of implicit bias would enable a holistic evaluation and provide clarity on where to apply targeted anti-bias interventions along each transitional milestone, a necessary objective to mitigate long-term health inequities.

## Data Availability

No datasets were generated or analysed during the current study.

## References

[1] FitzGerald C, Hurst S. Implicit bias in healthcare professionals: a systematic review. BMC Med Ethics. 2017;18:19–19. 28249596 10.1186/s12910-017-0179-8 PMC5333436

[2] De Houwer J. Implicit Bias is behavior: a functional-cognitive perspective on implicit Bias. Perspect Psychol Sci. 2019;14(5):835–40. 31374177 10.1177/1745691619855638

[3] Croskerry P. From mindless to mindful practice–cognitive bias and clinical decision making. N Engl J Med. 2013;368:2445. 23802513 10.1056/NEJMp1303712

[4] Kawamoto KR, Davis MB, Duvernoy CS. Acute coronary syndromes: differences in men and women. Curr Atheroscler Rep. 2016;18:73. 27807732 10.1007/s11883-016-0629-7

[5] van Ryn M, Hardeman R, Phelan SM, et al. Medical school experiences associated with change in implicit racial bias among 3547 students: a medical student CHANGES study report. J Gen Intern Med. 2015;30:1748–56. 26129779 10.1007/s11606-015-3447-7 PMC4636581

[6] Shah HS, Bohlen J. Implicit Bias. [Updated 2023 Mar 4]. In: StatPearls. Treasure Island (FL): StatPearls Publishing; 2024 Jan-. Available from: https://www.ncbi.nlm.nih.gov/books/NBK589697/. 36944001

[7] Hall WJ, Chapman MV, Lee KM, Merino YM, Thomas TW, Payne BK, Eng E, Day SH, Coyne-Beasley T. Implicit Racial/Ethnic Bias Among Health Care Professionals and Its Influence on Health Care Outcomes: A Systematic Review. Am J Public Health. 2015Dec;105(12):e60-76. 10.2105/AJPH.2015.302903 PMC4638275 26469668

[8] Cooper LA, Johnson RDL, Ford DE, Steinwache DM, Powe NR. Patient-centered communication, ratings of care, and concordance of patient and physician race. Ann Intern Med. 2003;139:907–15. 14644893 10.7326/0003-4819-139-11-200312020-00009

[9] Hamberg K. Gender Bias in Medicine. Women’s Health. 2008;4(3):237–43. 10.2217/17455057.4.3.237 19072473

[10] Phelan SM, Dovidio JF, Puhl RM, et al. Implicit and explicit weight bias in a national sample of 4,732 medical students: the medical student CHANGES study. Obesity (Silver Spring). 2014;22:1201. 24375989 10.1002/oby.20687 PMC3968216

[11] Rubin R. Addressing medicine’s bias against patients who are overweight. JAMA. 2019;321:925–7. 30785613 10.1001/jama.2019.0048

[12] Hernandez R. Medical students’ implicit bias and the communication of norms in medical education. Teach Learn Med. 2018;30(1):112–7. 29240453 10.1080/10401334.2017.1359610

[13] Mehta LS, Beckie TM, DeVon HA, et al. Acute myocardial infarction in women: a scientific statement from the American Heart Association Circulation. 2016;133:916–47. 26811316 10.1161/CIR.0000000000000351

[14] Zestcott CA, Blair IV, Stone J. Examining the presence, consequences, and reduction of implicit Bias in health care: a narrative review. Group Process Intergroup Relat. 2016;19(4):528–42. 27547105 10.1177/1368430216642029 PMC4990077

[15] Greenwald AG, McGhee DE, Schwartz JL. Measuring individual differences in implicit cognition: the implicit association test. J Pers Soc Psychol. 1998;74(6):1464–80. 9654756 10.1037//0022-3514.74.6.1464

[16] Schimmack U. The Implicit Association Test: A Method in Search of a Construct. Perspect Psychol Sci. 2021;16(2):396–414. 10.1177/1745691619863798. 10.1177/1745691619863798 31647752

[17] Fazio RH, Olson MA. Implicit measures in social cognition research: their meaning and use. Annu Rev Psychol. 2003;5:327–297. 10.1146/annurev.psych.54.101601.145225 12172003

[18] Sabin JA. Tackling Implicit Bias in Health Care. N Engl J Med. 2022;387(2):105–7. 35801989 10.1056/NEJMp2201180 PMC10332478

[19] Stone J, Moskowitz GB. Non-conscious bias in medical decision making: what can be done to reduce it? Med Educ. 2011;45(8):768–76. 21752073 10.1111/j.1365-2923.2011.04026.x

[20] Gonzalez CM, Lypson ML, Sukhera J. Twelve tips for teaching implicit bias recognition and management. Med Teach. 2021;43(12):1368–73. 33556288 10.1080/0142159X.2021.1879378 PMC8349376

[21] Sukhera J, Watling C. A framework for integrating implicit bias recognition into health professions education. Acad Med. 2018;93(1):35–40. 28658015 10.1097/ACM.0000000000001819

[22] Sabin J, Nosek BA, Greenwald A, Rivara FP. Physicians’ implicit and explicit attitudes about race by MD race, ethnicity, and gender. J Health Care Poor Underserved. 2009Aug;20(3):896–913. 19648715 10.1353/hpu.0.0185 PMC3320738

[23] FitzGerald C, Martin A, Berner D, et al. Interventions designed to reduce implicit prejudices and implicit stereotypes in real world contexts: a systematic review. BMC Psychol. 2019;7:29. 31097028 10.1186/s40359-019-0299-7 PMC6524213

[24] Baethge C, Goldbeck-Wood S, Mertens S. SANRA—a scale for the quality assessment of narrative review articles. Res Integr Peer Rev. 2019;4:5. 10.1186/s41073-019-0064-8. 30962953 10.1186/s41073-019-0064-8 PMC6434870

[25] BMC Med Educ. 2019;19(325).Minturn MS, Martinez EI, Le T, Nokoff N, Fitch L, Little CE, Lee RS. Early intervention for lgbtq health: a 10-hour curriculum for preclinical health professions students. MedEdPortal. 2021;17:11072. 10.15766/mep_2374-8265.11072 PMC7809930 33473382

[26] Petty J, Metzl JM, Keeys MR. Developing and Evaluating an Innovative Structural Competency Curriculum for Pre-Health Students. J Med Humanit. 2017;38(4):459–71. 10.1007/s10912-017-9449-1. 10.1007/s10912-017-9449-1 PMC5688197 28573595

[27] Metzl JM, Petty J, Olowojoba OV. Using a structural competency framework to teach structural racism in pre-health education. Soc Sci Med. 2018;199:189–201. 10.1016/j.socscimed.2017.06.029. 10.1016/j.socscimed.2017.06.029 28689630

[28] Goyal R, Martin S, Garbarski D. Perceptions of Cultural Competency Among Premedical Undergraduate Students. Qual Inq. 2020;7:445–53. 10.1177/1077800415572391. 10.1177/2382120520934823 PMC7430074 32864455

[29] Munk N, Church A, Nemati D, Zabel S, Comer AR. Massage perceptions and attitudes of undergraduate pre-professional health sciences students: a cross-sectional survey in one U.S. university. BMC Complement Med Ther. 2020;20(1). 10.1186/s12906-020-03002-6. 10.1186/s12906-020-03002-6 PMC7346672 32641024

[30] Copeland DJ, Johnson P, Moore B. Effects of a service‐learning experience on health related students' attitudes toward the homeless. Nurs Forum. 2020;56(1):45–51. 10.1111/nuf.12510. 10.1111/nuf.12510 32964482

[31] Martínez-Arnau FM, López-Hernández L, Castellano-Rioja E, Botella-Navas M, Pérez-Ros P. Interventions to improve attitudes toward older people in undergraduate health and social sciences students. A systematic review and meta-analysis. Nurse Educ Today. 2022;110:105269. 10.1016/j.nedt.2022.105269. 10.1016/j.nedt.2022.105269 35063781

[32] Baker TK, Smith GS, Jacobs NN, Houmanfar R, Tolles R, Kuhls D, Piasecki M. A deeper look at implicit weight bias in medical students. Adv Health Sci Educ Theory Pract. 2017;22(4):889–900. 10.1007/s10459-016-9718-1. 10.1007/s10459-016-9718-1 27734175

[33] Pettit KE, Turner JS, Kindrat JK, Blythe GJ, Hasty GE, Perkins AJ, Ashburn‐Nardo L, Milgrom LB, Hobgood CD, Cooper DD. Effect of Socioeconomic Status Bias on Medical Student Patient Interactions Using an Emergency Medicine Simulation. AEM Educ Train. 2017;1(2):126–31. 10.1002/aet2.10022. 10.1002/aet2.10022 PMC6001723 30051022

[34] Phelan SM, Burke SE, Hardeman RR, et al. Medical school factors associated with changes in implicit and explicit bias against gay and lesbian people among 3492 graduating medical students. J Gen Intern Med. 2017;32(11):1193–201. 28766125 10.1007/s11606-017-4127-6 PMC5653554

[35] Sawning S, Steinbock S, Croley R, Combs R, Shaw A, Ganzel T. A first step in addressing medical education curriculum gaps in lesbian-, gay-, bisexual-, and transgender-related content: the university of Louisville lesbian, gay, bisexual, and transgender health certificate program. Educ health. 2017;30(2):108–14. 10.4103/efh.EfH_78_16 28928340

[36] Geller G, Watkins PA. Addressing Medical Students' Negative Bias Toward Patients With Obesity Through Ethics Education. AMA J Ethics. 2018;20(10):E948–959. 10.1001/amajethics.2018.948. 10.1001/amajethics.2018.948 30346923

[37] Lawrence C, Mhlaba T, Stewart KA, Moletsane R, Gaede B, Moshabela M. The Hidden Curricula of Medical Education: A Scoping Review. Acad Med. 2018;93(4):648–56. 10.1097/ACM.0000000000002004. 10.1097/ACM.0000000000002004 PMC5938158 29116981

[38] Leslie KF, Sawning S, Shaw MA, Martin LJ, Simpson RC, Stephens JE, Jones VF. Changes in medical student implicit attitudes following a health equity curricular intervention. Medical Teacher. 2018;40(4):372–8 Ogunyemi D. Defeating unconscious bias: the role of a structured, reflective, and interactive workshop. J Grad Med Educ. 2021;13(2):189–194. 29171321 10.1080/0142159X.2017.1403014

[39] Marion GS, Hairston JM, Davis SW, Kirk JK. Using Standardized Patient Assessments to Evaluate a Health Literacy Curriculum. Fam Med. 2018;50(1):52–7. 10.22454/FamMed.2018.539107. 10.22454/FamMed.2018.539107 29346690

[40] Gonzalez CM, Deno ML, Kintzer E, Marantz PR, Lypson ML, McKee MD. A Qualitative Study of New York Medical Student Views on Implicit Bias Instruction: Implications for Curriculum Development. J Gen Intern Med. 2019;34(5):692–8. 10.1007/s11606-019-04891-1. 10.1007/s11606-019-04891-1 PMC6502892 30993612

[41] Horst A, Schwartz BD, Fisher JA, Michels N, Van Winkle LJ. Selecting and Performing Service-Learning in a Team-Based Learning Format Fosters Dissonance, Reflective Capacity, Self-Examination, Bias Mitigation, and Compassionate Behavior in Prospective Medical Students. Int J Environ Res Public Health. 2019;16(20):3926. 10.3390/ijerph16203926. 10.3390/ijerph16203926 PMC6843913 31623072

[42] Motzkus C, Wells RJ, Wang X, Chimienti S, Plummer D, Sabin J, Allison J, Cashman S. Pre-clinical medical student reflections on implicit bias: Implications for learning and teaching. PLOS ONE. 2019;14(11):e0225058. 10.1371/journal.pone.0225058. 10.1371/journal.pone.0225058 PMC6857943 31730651

[43] Phelan SM, Burke SE, Cunningham BA, Perry SP, Hardeman RR, Dovidio JF, Herrin J, Dyrbye LN, White RO, Yeazel MW, Onyeador IN, Wittlin NM, Harden K, van Ryn M. The Effects of Racism in Medical Education on Students’ Decisions to Practice in Underserved or Minority Communities. Acad Med. 2019;94(8):1178–89. 10.1097/ACM.0000000000002719. 10.1097/ACM.0000000000002719 30920443

[44] Acholonu RG, Cook TE, Roswell RO, Greene RE. Interrupting Microaggressions in Health Care Settings: A Guide for Teaching Medical Students. MedEdPORTAL. 10.15766/mep_2374-8265.10969. 10.15766/mep_2374-8265.10969 PMC7394346 32754633

[45] Benoit LJ, Travis C, Swan Sein A, Quiah SC, Amiel J, Gowda D. Toward a Bias-Free and Inclusive Medical Curriculum: Development and Implementation of Student-Initiated Guidelines and Monitoring Mechanisms at One Institution. Acad Med. 2020;95(12S):S145–S149. 10.1097/ACM.0000000000003701. 10.1097/ACM.0000000000003701 32889934

[46] Gonzalez CM, Walker SA, Rodriguez N, Karp E, Marantz PR. It Can Be Done! A Skills-Based Elective in Implicit Bias Recognition and Management for Preclinical Medical Students. Acad Med. 2020;95(12S):S150–S155. 10.1097/ACM.0000000000003697. 10.1097/ACM.0000000000003697 PMC7686093 32889927

[47] Morris MC, Cooper RL, Ramesh A, Tabatabai M, Arcury TA, Shinn M, Im W, Juarez Paul, Matthews-Juarez P. Preparing Medical Students to Address the Needs of Vulnerable Patient Populations: Implicit Bias Training in US Medical Schools Med Sci Educ. 2020;30(1):123–7. 10.1007/s40670-020-00930-3. 10.1007/s40670-020-00930-3 PMC8368413 34457650

[48] Ona FF, Amutah-Onukagha NN, Asemamaw R, Schlaff AL. Struggles and Tensions in Antiracism Education in Medical School: Lessons Learned. Acad Med. 2020;95(12S):S163–S168. 10.1097/ACM.0000000000003696. 10.1097/ACM.0000000000003696 33229958

[49] Rivlin K, Sedlander E, Cepin A. "It Allows You to Challenge Your Beliefs": Examining Medical Students' Reactions to First Trimester Abortion. Womens Health Issues. 2020;30(5):353–8. 10.1016/j.whi.2020.06.004. 10.1016/j.whi.2020.06.004 32669243

[50] Ruben M, Saks NS. Addressing Implicit Bias in First-Year Medical Students: a Longitudinal Multidisciplinary Training Program. Med Sci Educ. 2020;30(4):1419–26. 10.1007/s40670-020-01047-30. 10.1007/s40670-020-01047-3 PMC8368581 34457809

[51] Fitterman-Harris HF, Vander Wal JS. Weight bias reduction among first-year medical students: A quasi-randomized, controlled trial. Clin Obes. 2021;11(6):e12479. 10.1111/cob.12479. 10.1111/cob.12479 34263533

[52] Gonzalez CM, Noah YS, Correa N, Archer‐Dyer H, Weingarten‐Arams J, Sukhera J. Qualitative analysis of medical student reflections on the implicit association test. Med Educ. 2021;55(6):741–8. 10.1111/medu.14468. 10.1111/medu.14468 PMC8119345 33544914

[53] Gonzalez CM, Walker SA, Rodriguez N, Noah YS, Marantz Paul R. Implicit Bias Recognition and Management in Interpersonal Encounters and the Learning Environment: A Skills-Based Curriculum for Medical Students. MedEdPORTAL. 10.15766/mep_2374-8265.11168. 10.15766/mep_2374-8265.11168 PMC8275619 34277934

[54] Nestorowicz S, Saks N. Addressing Bias Toward Overweight Patients: a Training Program for First-Year Medical Students. Med Sci Educ. 2021;31(3):1115–23. 10.1007/s40670-021-01282-2. 10.1007/s40670-021-01282-2 PMC8368903 34457955

[55] Phelan SM, Puhl RM, Burgess DJ, Natt N, Mundi M, Miller NE, Saha S, Fischer K, van Ryn M. The role of weight bias and role-modeling in medical students' patient-centered communication with higher weight standardized patients. Patient Educ Couns. 2021;104(8):1962–9. 10.1016/j.pec.2021.01.003. 10.1016/j.pec.2021.01.003 33487507

[56] Van Winkle LJ, Schwartz BD, Horst A, Fisher JA, Michels N, Thornock BO. Impact of a Pandemic and Remote Learning on Team Development and Elements of Compassion in Prospective Medical Students Taking a Medical Humanities Course. Int J Environ Res Public Health. 2021;18(9):4856. 10.3390/ijerph18094856. 10.3390/ijerph18094856 PMC8124650 34063219

[57] Matsumoto MM, Schultz O, Jiang T, Navuluri R. Recruitment in Surgery and Interventional Radiology: Factors in Female Trainees' Specialty Decisions. J Surg Educ. 2020;77(6):1454–64. 10.1016/j.jsurg.2020.06.002. 10.1016/j.jsurg.2020.06.002 32571694

[58] Chen S, Beck Dallaghan GL, Shaheen A. Implicit Gender Bias in Third-Year Surgery Clerkship MSPE Narratives. J Surg Educ. 2021;78(4):1136–43. 10.1016/j.jsurg.2020.10.011. 10.1016/j.jsurg.2020.10.011 33129771

[59] Gopal DP, Chetty U, O'Donnell P, Gajria C, Blackadder-Weinstein J. Implicit bias in healthcare: clinical practice, research and decision making. Future Healthc J. 2021;8(1):40–8. 10.7861/fhj.2020-0233. 10.7861/fhj.2020-0233 PMC8004354 33791459

[60] Fassiotto M, Li J, Maldonado Y, Kothary N. Female Surgeons as Counter Stereotype: The Impact of Gender Perceptions on Trainee Evaluations of Physician Faculty. J Surg Educ. 2018;75(5):1140–8. 10.1016/j.jsurg.2018.01.011. 10.1016/j.jsurg.2018.01.011 29402668

[61] Halvorson EE, Curley T, Wright M, Skelton JA. Weight Bias in Pediatric Inpatient Care. Acad Pediatr. 2019;19(7):780–6. 10.1016/j.acap.2019.02.005. 10.1016/j.acap.2019.02.005 PMC6703967 30796998

[62] Morris M, Cooper RL, Ramesh A, et al. Training to reduce LGBTQ-related bias among medical, nursing, and dental students and providers: a systematic review. BMC Med Educ. 2019;19:325. 10.1186/s12909-019-1727-3. 31470837 10.1186/s12909-019-1727-3 PMC6716913

[63] Brottman MR, Char DM, Hattori RA, Heeb R, Taff SD. Toward cultural competency in health care: a scoping review of the diversity and inclusion education literature. Acad Med. 2020;95(5):803–13. 31567169 10.1097/ACM.0000000000002995

[64] Mastrocola MR, Roque SS, Benning LV, Stanford FC. Obesity education in medical schools residencies and fellowships throughout the world: a systematic review. Int J Obes. 2020;44(2):269–79. 10.1038/s41366-019-0453-6. 10.1038/s41366-019-0453-6 PMC7002222 31551484

[65] Teherani A, Perez S, Muller-Juge V, Lupton K, Hauer KE. A Narrative Study of Equity in Clinical Assessment Through the Antideficit Lens. Acad Med. 2020;95(12S):S121–S130. 10.1097/ACM.0000000000003690. 10.1097/ACM.0000000000003690 33229956

[66] Tobon AL, Budde KS, Rohrbaugh RM. A Novel Approach to Fostering Diversity in Graduate Medical Education: Chief Residents for Diversity and Inclusion. Acad Psychiatry. 2019;43(3):344–5. 10.1007/s40596-019-01055-5. 10.1007/s40596-019-01055-5 31041660

[67] Ogunyemi D. Defeating Unconscious Bias: The Role of a Structured, Reflective, and Interactive Workshop. J Grad Med Educ. 2021;13(2):189-94. 10.4300/JGME-D-20-00722.1. 10.4300/JGME-D-20-00722.1 PMC8054602 33897951

[68] Xiong M, Young AT, Bray SMC. A survey of gender-based barriers and misconceptions in surgery. Surg Pract Sci. 2022;8:100048. 10.1016/j.sipas.2021.100048. https://www.sciencedirect.com/science/article/pii/S2666262021000243.

[69] Bartlett K, Strelitz P, Hawley J, et al. Explicitly Addressing Implicit Bias in a Cultural Competence Curriculum for Pediatric Trainees [version 1]. MedEdPublish. 2019;8:102. 10.15694/mep.2019.000102.1.

[70] Kallianos KG, Webb EM, Hess CP, Talbott J, Bucknor MD. Use of the Implicit Association Test to Improve Diversity in Radiology. J Am Coll Radiol. 2019;16(7):976–9. 10.1016/j.jacr.2019.01.010. 10.1016/j.jacr.2019.01.010 30982682

[71] Sherman MD, Ricco J, Nelson SC, Nezhad SJ, Prasad S. Implicit Bias Training in a Residency Program: Aiming for Enduring Effects. Fam Med. 2019;51(8):677–81. 10.22454/FamMed.2019.947255. 10.22454/FamMed.2019.947255 31509218

[72] Herr KD, George E, Agarwal V, McKnight CD, Jiang L, Jawahar A, Pakkal M, Ulano A, Ganeshan D. Aligning the Implicit Curriculum with the Explicit Curriculum in Radiology. Acad Radiol. 2020;27(9):1268–73. 10.1016/j.acra.2019.12.028. 10.1016/j.acra.2019.12.028 32061468

[73] Perdomo J, Tolliver D, Hsu H, He Y, Nash KA, Donatelli S, Mateo C, Akagbosu C, Alizadeh F, Power-Hays A, Rainer T, Zheng DJ, Kistin CJ, Vinci RJ, Michelson CD. Health Equity Rounds: An Interdisciplinary Case Conference to Address Implicit Bias and Structural Racism for Faculty and Trainees. MedEdPORTAL. 10.15766/mep_2374-8265.10858. 10.15766/mep_2374-8265.10858 PMC7050660 32166114

[74] Barnes KL, McGuire L, Dunivan G, Sussman AL, McKee R. Gender Bias Experiences of Female Surgical Trainees. J Surg Educ. 2019;76(6):e1–e14. 10.1016/j.jsurg.2019.07.024. https://www.sciencedirect.com/science/article/pii/S1931720419303058. 10.1016/j.jsurg.2019.07.024 31601487

[75] Johnson TJ, Winger DG, Hickey RW, Switzer GE, Miller E, Nguyen MB, Saladino RA, Hausmann LRM. Comparison of Physician Implicit Racial Bias Toward Adults Versus Children. Acad Pediatr. 2017;17(2):120–6. 10.1016/j.acap.2016.08.010. 10.1016/j.acap.2016.08.010 PMC5337439 27620844

[76] Kulaylat AN, Qin D, Sun SX, Hollenbeak CS, Schubart JR, Aboud AJ, Flemming DJ, Bollard ER, Dillon PW, Han DC. Aligning perceptions of mistreatment among incoming medical trainees. J Surg Res. 2017;208:151–7. 10.1016/j.jss.2016.09.016. 10.1016/j.jss.2016.09.016 27993202

[77] Chapman MV, Hall WJ, Lee K, Colby R, Coyne-Beasley T, Day S, Eng E, Lightfoot AF, Merino Y, Simán FM, Thomas T, Thatcher K, Payne K. Making a difference in medical trainees' attitudes toward Latino patients: A pilot study of an intervention to modify implicit and explicit attitudes. Soc Sci Med. 2018;199:202–8. 10.1016/j.socscimed.2017.05.013. 10.1016/j.socscimed.2017.05.013 PMC5714690 28532893

[78] Bucknor MD, Villanueva-Meyer JE, Kumar V, Talbott JF, Wall SD, Glastonbury CM, Dillon WP, Arenson RL, Wilson MW, Hess CP. Diversity and Inclusion Efforts in University of California, San Francisco Radiology: Reflections on 3 Years of Pipeline, Selection, and Education Initiatives. J Am Coll Radiol. 2019;16(12):1716–9. 10.1016/j.jacr.2019.06.012. 10.1016/j.jacr.2019.06.012 31299250

[79] Dyrbye L, Herrin J, West CP, Wittlin NM, Dovidio JF, Hardeman R, Burke SE, Phelan S, Onyeador IN, Cunningham B, van Ryn M. Association of Racial Bias With Burnout Among Resident Physicians. JAMA Netw Open. 2019;2(7):e197457. 10.1001/jamanetworkopen.2019.7457. 10.1001/jamanetworkopen.2019.7457 PMC6661712 31348503

[80] Gerull KM, Loe M, Seiler K, McAllister J, Salles A. Assessing gender bias in qualitative evaluations of surgical residents. Am J Surg. 2019;217(2):306–13. 10.1016/j.amjsurg.2018.09.029. https://www.sciencedirect.com/science/article/pii/S0002961018306317. 10.1016/j.amjsurg.2018.09.029 PMC8687875 30343879

[81] Hansen M, Schoonover A, Skarica B, Harrod T, Bahr N, Guise J-M. Implicit gender bias among US resident physicians. BMC Med Educ. 2019;19(1). 10.1186/s12909-019-1818-1. 10.1186/s12909-019-1818-1 PMC6819402 31660944

[82] Khatri U, Zeidan A, LaRiviere M, et al. An Evaluation of Implicit Bias Training in Graduate Medical Education. MedEdPublish. 2019. 10.15694/mep.2019.000109.1.

[83] Klein R, Law K, Koch J. Gender Representation Matters: Intervention to Solicit Medical Resident Input to Enable Equity in Leadership in Graduate Medical Education. Acad Med. 2020;95(12S):S93–S97. 10.1097/ACM.0000000000003698. 10.1097/ACM.0000000000003698 32889942

[84] Lukela JR, Ramakrishnan A, Hadeed N, et al. When perception is reality: Resident perception of faculty gender parity in a university-based internal medicine residency program. Perspect Med Educ. 2019;8:346–52. 10.1007/s40037-019-00532-9. 10.1007/s40037-019-00532-9 PMC6904409 31728840

[85] McKinley SK, Wang LJ, Gartland RM, Westfal ML, Costantino CL, Schwartz D, Merrill AL, Petrusa E, Lillemoe K, Phitayakorn R; Massachusetts General Hospital Gender Equity Task Force. "Yes, I'm the Doctor": One Department's Approach to Assessing and Addressing Gender-Based Discrimination in the Modern Medical Training Era. Acad Med. 2019;94(11):1691–8. 10.1097/ACM.0000000000002845. 10.1097/ACM.0000000000002845 31274522

[86] Wittlin NM, Dovidio JF, Burke SE, Przedworski JM, Herrin J, Dyrbye L, Onyeador IN, Phelan SM, van Ryn M. Contact and role modeling predict bias against lesbian and gay individuals among early-career physicians: A longitudinal study. Soc Sci Med. 2019;238:112422. 10.1016/j.socscimed.2019.112422. 10.1016/j.socscimed.2019.112422 PMC6744977 31391147

[87] Zeidan AJ, Khatri UG, Aysola J, Shofer FS, Mamtani M, Scott KR, Conlon LW, Lopez BL. Implicit Bias Education and Emergency Medicine Training: Step One? Awareness. AEM Educ Train. 2018;3(1):81–5. 10.1002/aet2.10124. 10.1002/aet2.10124 PMC6339553 30680351

[88] Kassam AF, Cortez AR, Winer LK, Baker JE, Hanseman DJ, Wells D, Yalamanchili S, Habashy E, Chausse S, Makley AT, Goodman MD, Sussman JJ, Quillin RC 3rd. Swipe right for surgical residency: Exploring the unconscious bias in resident selection. Surgery. 2020;168(4):724–9. 10.1016/j.surg.2020.05.029. 10.1016/j.surg.2020.05.029 32675032

[89] Klein R, Law K, Koch J. Gender Representation Matters: Intervention to Solicit Medical Resident Input to Enable Equity in Leadership in Graduate Medical Education. Acad Med. 2020;95(12S Addressing Harmful Bias and Eliminating Discrimination in Health Professions Learning Environments):S93–S97. 10.1097/ACM.0000000000003698. 10.1097/ACM.0000000000003698 32889942

[90] Kuo LE, Lyu HG, Jarman MP, Melnitchouk N, Doherty GM, Smink DS, Cho NL. Gender Disparity in Awards in General Surgery Residency Programs. JAMA Surg. 2020;156(1):60–6. 10.1001/jamasurg.2020.3518. 10.1001/jamasurg.2020.3518 PMC7489428 32876660

[91] Sabin J, Calista J, Dykhouse E, Eisdorfer E, Foiles A, Garcia M, Hale J, Puerto G, Rappaport L, Terrien J, Valdman O, Yazdani M, Tjia J. Minimizing Defensiveness in Clinician Education about Implicit Bias: Lessons Learned from a Community‐Engaged Randomized Clinical Trial. Health Serv Res. 2020;55(S1):51–2. 10.1111/1475-6773.13399.

[92] Thomas B, Booth-McCoy AN. Blackface, Implicit Bias, and the Informal Curriculum: Shaping the Healthcare Workforce, and Improving Health. J Natl Med Assoc. 2020;112(5):533–40. 10.1016/j.jnma.2020.05.012. 10.1016/j.jnma.2020.05.012 32646723

[93] Barber Doucet H, Ward VL, Johnson TJ, Lee LK. Implicit Bias and Caring for Diverse Populations: Pediatric Trainee Attitudes and Gaps in Training. Clin Pediatr (Phila). 2020;60(9-10):408–17. 10.1177/00099228211035225. 10.1177/00099228211035225 34308661

[94] Chatterjee P, Warner LN, Basil MC, Christopher M, Manning K, Fisher HN, Rexrode KM, Solomon SR, Kakoza RM, Yialamas MA. "Make the Implicit Explicit": Measuring Perceptions of Gender Bias and Creating a Gender Bias Curriculum for Internal Medicine Residents. Adv Med Educ Pract. 2021;12:49–52. 10.2147/AMEP.S292166. 10.2147/AMEP.S292166 PMC7814655 33488136

[95] Dill-Macky A, Hsu C-H, Neumayer LA, Nfonsam VN, Turner AP. The Role of Implicit Bias in Surgical Resident Evaluations. J Surg Educ. 2022;79(3):761–8. 10.1016/j.jsurg.2021.12.003. https://www.sciencedirect.com/science/article/pii/S1931720421003470. 10.1016/j.jsurg.2021.12.003 34973900

[96] Kramer M, Heyligers IC, Könings KD. Implicit gender-career bias in postgraduate medical training still exists, mainly in residents and in females. BMC Med Educ. 2021;21(1):253. 10.1186/s12909-021-02694-9. 10.1186/s12909-021-02694-9 PMC8088689 33933035

[97] Ouyang K, Huang IA, Wagner JP, Wu J, Chen F, Quach C, Donahue TR, Hines OJ, Hiatt JR, Tillou A. Persistence of Gender Bias Over Four Decades of Surgical Training. J Surg Educ. 2021;78(6):1868–77. 10.1016/j.jsurg.2021.06.008. 10.1016/j.jsurg.2021.06.008 34294569

[98] Roth LT, Catallozzi M, Soren K, Lane M, Friedman S. Bridging the gap in graduate medical education: a longitudinal pediatric lesbian, gay, bisexual, transgender, queer/questioning health curriculum. Acad Pediatr. 2021;21(8):1449–57. 34098174 10.1016/j.acap.2021.05.027

[99] Burke SE, Dovidio JF, Przedworski JM, Hardeman RR, Perry SP, Phelan SM, Van Ryn M. Do contact and empathy mitigate bias against gay and lesbian people among heterosexual first-year medical students? A report from the medical student CHANGE study. Academic Medicine. 2015;90(5):645–51. 25674910 10.1097/ACM.0000000000000661 PMC4414697

[100] Hemphill ME, Maher Z, Ross HM. Addressing Gender-Related Implicit Bias in Surgical Resident Physician Education: A Set of Guidelines. J Surg Educ. 2020;77(3):491–4. 10.1016/j.jsurg.2019.12.014. Epub 2020 Jan 16. PMID: 31954662. 31954662 10.1016/j.jsurg.2019.12.014

[101] Morris M, Cooper RL, Ramesh A, et al. Training to reduce LGBTQ-related bias among medical, nursing, and dental students and providers: a systematic review. BMC Med Educ. 2019;19:325. 10.1186/s12909-019-1727-3. 10.1186/s12909-019-1727-3 PMC6716913 31470837

[102] Yazdani S, Andarvazh MR, Afshar L. What is hidden in hidden curriculum? a qualitative study in medicine. J Med Ethics Hist Med. 2020May;10(13):4. 10.18502/jmehm.v13i4.2843 PMC7569532 33088431

[103] Gonzaled CM, Walker SA, Rodriguez N, Noah YS, Marantz PR. Implicit bias recognition and management in interpersonal encounters and the learning environment: a skills-based curriculum for medical students. MedEdPortal. 2021;13(17):11168. 10.15766/mep_2374-8265.11168 PMC8275619 34277934

[104] Norman Geoffrey R, Monteiro Sandra D, Sherbino Jonathan, Ilgen Jonathan S, Schmidt Henk G, Mamede Silvia. The Causes of Errors in Clinical Reasoning: Cognitive Biases, Knowledge Deficits, and Dual Process Thinking. Academic Medicine. 2017;92(1):23–30. 27782919 10.1097/ACM.0000000000001421

[105] Wright JL, Davis WS, Joseph MM, Ellison AM, Heard-Garris NJ, Johnson TL; AAP Board Committee on Equity. Eliminating Race-Based Medicine. Pediatrics. 2022;150(1):e2022057998. 10.1542/peds.2022-057998. 10.1542/peds.2022-057998 35491483

